# Differences in clinical severity of respiratory viral infections in hospitalized children

**DOI:** 10.1038/s41598-021-84423-2

**Published:** 2021-03-04

**Authors:** Benjamin M. Althouse, Stefan Flasche, Michiko Toizumi, Hien-Anh Thi Nguyen, Hien Minh Vo, Minh Nhat Le, Masahiro Hashizume, Koya Ariyoshi, Dang Duc Anh, Gail L. Rodgers, Keith P. Klugman, Hao Hu, Lay-Myint Yoshida

**Affiliations:** 1 Institute for Disease Modeling, 3150 139th Ave SE, Bellevue, WA 98005 USA; 2 University of Washington, Seattle, WA USA; 3 New Mexico State University, Las Cruces, NM USA; 4 London School of Hygiene and Tropical Medicine, London, UK USA; 5 Institute of Tropical Medicine, Nagasaki University, Nagasaki, Japan; 6 National Institute of Hygiene and Epidemiology, Hanoi, Vietnam; 7 Khanh Hoa General Hospital, Nha Trang, Vietnam; 8 Bill & Melinda Gates Foundation, Seattle, WA USA

**Keywords:** Viral infection, Respiratory signs and symptoms, Paediatrics

## Abstract

It is uncertain whether clinical severity of an infection varies by pathogen or by multiple infections. Using hospital-based surveillance in children, we investigate the range of clinical severity for patients singly, multiply, and not infected with a group of commonly circulating viruses in Nha Trang, Vietnam. RT-PCR was performed to detect 13 respiratory viruses in nasopharyngeal samples from enrolled patients. We apply a novel clinical severity score and examine associations with the odds of being severe and differences in raw severity scores. We find no difference in severity between 0-, 1-, and 2-concurrent infections and little differences in severity between specific viruses. We find RSV and HMPV infections to be associated with 2- and 1.5-fold increase in odds of being severe, respectively, and that infection with ADV is consistently associated with lower risk of severity. Clinically, based on the results here, if RSV or HMPV virus is suspected, PCR testing for confirmatory diagnosis and for detection of multiple coinfecting viruses would be fruitful to assess whether a patient’s disease course is going to be severe.

## Introduction

Acute respiratory infections (ARIs) cause substantial morbidity and mortality across the globe, especially in those under 5 years of age^1^. For a clinician, rapid identification of the potential clinical severity of an incoming patient becomes paramount, especially in resource-limited settings and high-volume health care facilities. Thus, with many different pathogens circulating within a community, it becomes important to understand what differences exist in patient severity by infecting pathogen and by whether the patient is infected with one, two, or more viruses simultaneously. While several studies have examined the severity of single versus $>1$ infections and found no differences in multiple severity indicators (as reviewed in Scotta et al.^2^, Lim et al.^3^, and Asner et al.^4^), little is known about potential differences in clinical severity across specific viruses in single versus multiply-infected individuals.

Previous work is equivocal on the severity of respiratory syncytial virus (RSV) and human metapneumo virus (HMPV) coinfections. Some reports indicate increased severity in RSV and HMPV coinfected individuals^5–7^, while others do not^8,9^. Several studies have compared the severity of Influenza A (Flu A) and B (Flu B) coinfections: Goka et al.^10^ found increased hospitalization rates for Flu A/B coinfected individuals, while Rhedin et al.^11^ found no increase in clinical severity for Flu A H1N1 coinfected individuals. Fewer studies have focused on the role of coinfection with other common, and less common respiratory viruses including adenovirus (ADV)^12^, human bocavirus (HBoV)^13^, human parainfluenza viruses (HPIV1-4)^2^, and human rhinovirus (HRV)^14^. Knowledge of differing clinical severity of these pathogens in mono and multiply infected individuals is lacking.

Finally, most of the reports of virus-specific and coinfection severity have not been conducted in developing countries. A systematic review of multiple respiratory infections and severity thereof by Scotta et al. reported 6 studies from China, 3 from Vietnam, and 1 each from Cambodia and Madagascar^2^. Thus, it is the goal of the present study to explore indicators of clinical severity, including the role of specific viruses and 0, 1, and multiple concurrent infections, in a large cohort of hospitalized children in Nha Trang, Vietnam.

## Methods

### Study site

The study site is Nha Trang, central Vietnam, where the study population has been described previously^15–17^.The ongoing study was initiated as an active prospective surveillance study of respiratory illness in children under the age of 16 years in Nha Trang, enroll all hospitalized ARI cases from our target population. According to the field site census survey in July 2006, the study catchment area of 16 communes in Nha Trang city, had 198,729 residents including 13,952 children less than 5 years of age. We analyze data from January 29, 2007 to April 26, 2012 at Khanh Hoa General Hospital (KHGH) which is the only tertiary care facility located in Khanh Hoa Province and the only one accessible for residents of the catchment area. An ARI case was defined as any child presenting to KHGH with cough or/and difficulty in breathing. Eligible children were under 16 years old and were enrolled at all times of the year. Clinical and demographic information, chest radiographs (CXR), laboratory data, and nasopharyngeal (NP) samples were collected from all enrolled patients. Radiographically-confirmed pneumonia (RCP) was categorized using the WHO standard case definition^18^. In 2013 all CXRs were re-read independently by two designated radiologists. An expert panel reviewed discordant readings and a sample of concurrent readings. Acute respiratory infection patients with normal CXR were categorized as upper respiratory tract infection (URTI). Patients with abnormal CXR were categorized as lower respiratory tract infection (LRTI).

Laboratory methods have been previously described^16^. NP swabs were collected at the time of admission and viral nucleic acid was extracted using QIA viral RNA minikit (QIAGEN Inc., Valencia, CA). Four multiplex-PCR assays (1: influenza A, influenza B, RSV, hMPV; 2: HPIV-1, -2, -3, and -4; 3: rhinovirus, coronavirus 229E, coronavirus OC43; 4: adenovirus and bocavirus) were performed to detect 13 respiratory viruses in each NP sample. A second confirmatory-PCR was performed for samples positive on the initial PCR test. Samples positive for both PCR assays were defined as positive. The samples were screened for 13 respiratory viruses using four multiplex PCR assays. The positive PCR product will be used to conduct a second PCR (heminested PCR) with inner primers targeting the respective gene of the virus. This two step screening and confirmation can detect target respiratory viruses with 1000–10,000 copies per reaction. The assay has a high specificity that only less than 1% of the samples positive in first assay will be negative with the second nested confirmatory PCR.

### Ethics, consent and permissions

The authors assert that all of the procedures contributing to this work comply with the ethical standards of the relevant national and institutional committees on human experimentation and the Helsinki Declaration of 1975 as revised in 2008. Before study enrollment, informed consent was obtained from parents of children who presented with ARI and lived in the study catchment area. The study was approved by institutional review boards in the National Institute of Hygiene and Epidemiology, Vietnam and the Institute of Tropical Medicine, Nagasaki University, Japan.

### Indicators of clinical severity

We modified a clinical severity score (CSS) developed previously^19,20^ to include additional variables collected in our study. The original CSS gave 2 points if the child required mechanical ventilation during their hospitalization, and 1 point for each of the following: hospital admission (all children), hospitalization for 5 or more days, oxygen saturation less than 87%, and use of supplemental oxygen. In addition to this, we add 1 point each if the child has chest indrawing, wheezing, tachypnea, or difficulty breathing. Our score ranges from 0 to 10. We define severe respiratory disease as children with CSS $> 3$. We explore cutoffs of 2 and 4 in the supplementary material. In addition, wheezing has been associated with less severe LRTI in children without danger signs such as chest indrawing or malnutrition^21^ and equivocally with RSV infection^22^, thus in the supplement we explore the associations between virus detections and wheezing and present results of the risk score without wheezing. The CSS was calculated once for each child retroactively with scores calculated with data from the entire hospital stay. Future studies should calculate the score upon admission.

### Statistical analysis

We compare the CSS across viruses using means and interquartile ranges (IQR) and calculate multinomial confidence intervals using the method of Sison and Graz^23^, as implemented in the R package MultinomialCI^24^. We use one- and two-sided proportion tests to compare the proportion of severe disease by virus and mono-, multiply-infected, and PCR negative patients and Wilcoxon signed rank tests to compare raw CSS. Logistic regression models were formed with severe disease (CSS $>3$) as the outcome and the individual virus as the predictor. We fit single logistic regressions for each virus and multiple logistic regressions to adjust for age, sex, smoking inside the child’s home, socioeconomic status (SES), calendar year, month of year, daycare attendance, and breastfeeding status. We also form logistic regressions to compare the odds of severe disease by number of viruses detected (0, 1, 2, $\ge 3$). To compare the differences in CSS between viruses, we formulate single linear regressions and multiple linear regressions (adjusted for the variables listed above) with CSS score as the outcome and the presence/absence of each virus as a predictor. All analyses were conducted in R 3.3.1^25^

### Sensitivity analyses

Sensitivity analyses, presented in the supplementary materials, include changing the CSS cutoff for classifying a patient as severe to $>2$ and $>4$, as well as Bayesian logistic regressions to account for potential sparse data biases^26^, and examination of severity in children with no known underlying conditions.

## Results

### Predictors of severity

**Figure 1:**
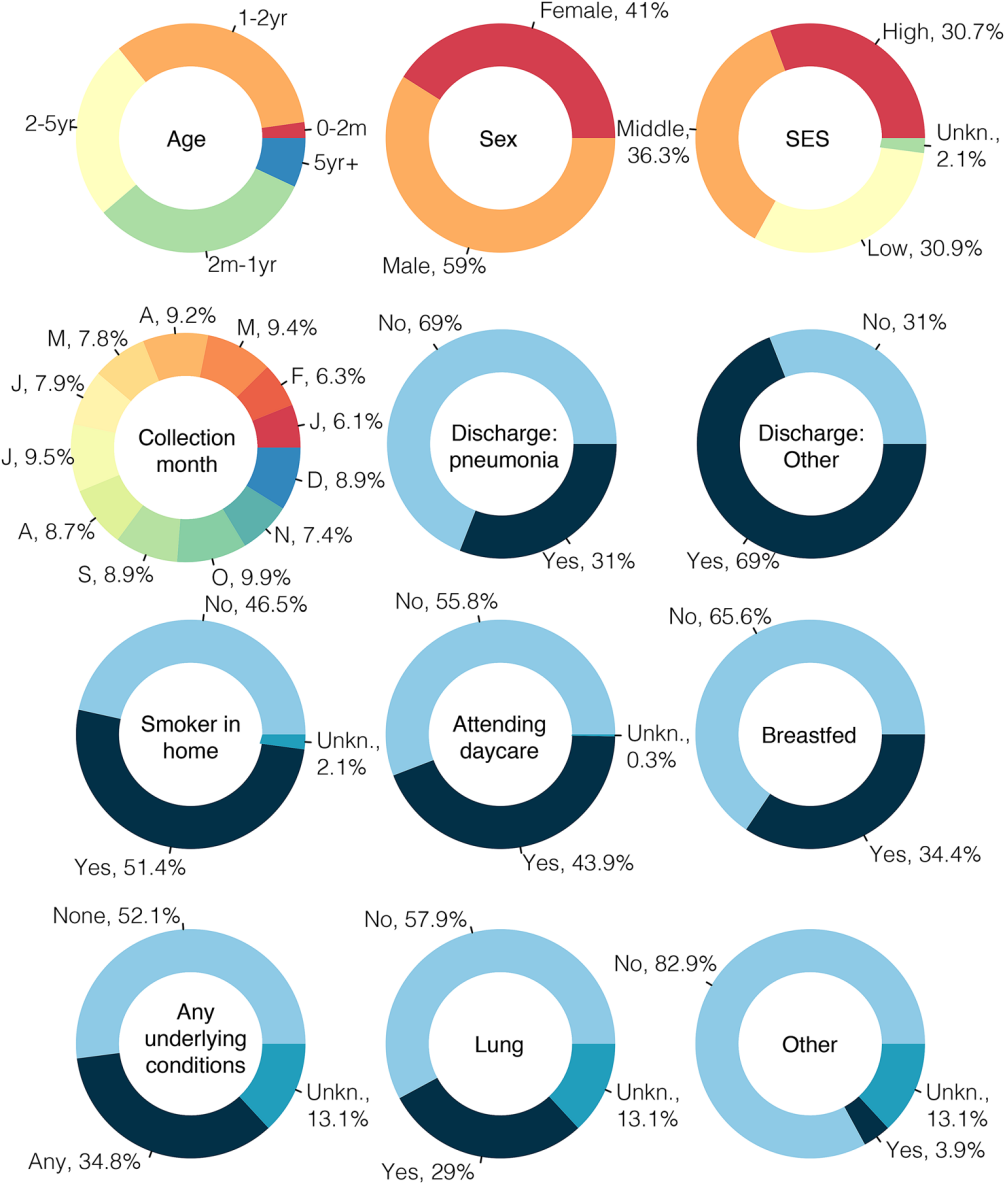
Demographics of the study cohort. Figure shows the demographics of the cohort. Underlying conditions of chronic diarrhea, cardiac, neurological, renal, malnourished, and thalassemia had less than 5% prevalences each. Similarly with discharges of rhinitis, pharyngitis, tonsillitis, otitis media, croup, trach, bronchitis, meningitis, and sepsis had prevalences less than 5%. We note that the colors are arbitrary. SES, socioeconomic status.

The study enrolled 3431 children between 2007 and 2013. Among those, 28 did not have clinical samples or RCP screen and were excluded from the analyses, thus the total study population was 3403. The age of hospitalized children ranged from 14 days to 15 years, with a median of 17 months (interquartile range [IQR]: 9, 28). Number of virus detections ranged from 0 to 4 detections (Table 1; Fig. 1). CSS scores were generally low, the vast majority of CSS were 1 and 2 (2271 of 3403, 67%), though 33% (1132 of 3403) were classified as severe cases (CSS $>3$), and 285 patients (8.4%) had scores over 5. Multivariate logistic regression showed male sex (OR 1.36 [95% CI 1.1, 1.7, $p= 0.005$) as a positive predictor of clinical severity (Table 2). No association was found between RCP and odds of being classified as a severe case (OR 1.35 [95% CI 0.84, 2.08, p = 0.191]). RCP was significantly associated with a higher raw CSS (2.64 [95% CI 2.43, 2.84] vs. 2.29 [95% CI 2.25, 2.33]; Wilcoxon signed rank: $p<0.001$). Small, but significant differences in CSS were seen between 0–12, 12–24, and over 24 months of age (2.47 [95% CI 2.39, 2.54], 2.3 [95% CI 2.23, 2.38], and 2.15 [95% CI 2.08, 2.22] vs. 2.01 [95% CI 1.89, 2.13]; Kruskal–Wallis rank sum test, $p<0.001$).

**Table 1:** Numbers of virus detections by age.

Age	Detections (% by age)					
	0	1	2	3	4	Total
0–6 months	175 (37.8%)	246 (53.1%)	39 (8.4%)	3 (0.6%)	0 (0%)	465
6 months–1 year	390 (34.3%)	578 (50.9%)	147 (12.9%)	19 (1.7%)	2 (0.2%)	1138
1–2 year	361 (41.5%)	429 (49.4%)	70 (8.1%)	7 (0.8%)	2 (0.2%)	871
2–5 year	115 (49.4%)	115 (49.4%)	3 (1.3%)	0 (0%)	0 (0%)	233
5 year+	275 (39.6%)	343 (49.4%)	67 (9.7%)	9 (1.3%)	0 (0%)	696
Total	1316 (38.7%)	1711 (50.3%)	326 (9.6%)	38 (1.1%)	4 (0.1%)	3403

Table gives the numbers of virus detections (0–4) by age for the study population. Percents are percents of age class by number of detection, ie, 175 of 465 0–6 month olds (37.8%) had 0 detections.

**Table 2:** Predictors of clinical severity.

Predictor	Multivariate OR (95% CI, p)
Coinfection	1.01 (0.72, 1.41, p = 0.932)
RCP	1.35 (0.84, 2.08, p = 0.191)
Age	1.16 (0.54, 2.35, p = 0.697)
Male	**1.36 (1.1, 1.69, p = 0.005)**
Smoke inside	0.97 (0.86, 1.08, p = 0.557)
Daycare	0.83 (0.65, 1.05, p = 0.124)
Breastfeeding	1.07 (0.83, 1.39, p = 0.602)
Low SES	1.08 (0.83, 1.4, p = 0.58)
Middle SES	1.02 (0.79, 1.32, p = 0.888)
High SES	Ref.

Table gives the adjusted odds ratios of being a severe clinical case (CSS $>3$) by coinfection status (more than 1 detection), RCP (yes/no), per year of age, Male sex (male vs. female), smoking in the home (yes/no), attending daycare (yes/no), breastfeeding status (yes/no), and socioeconomic status (SES; low and middle vs. high). The model is additionally adjusted for calendar year and month of year.

Estimates displayed in bold face indicate statistical significance (p<0.05).

### Severity across viruses

**Figure 2:**
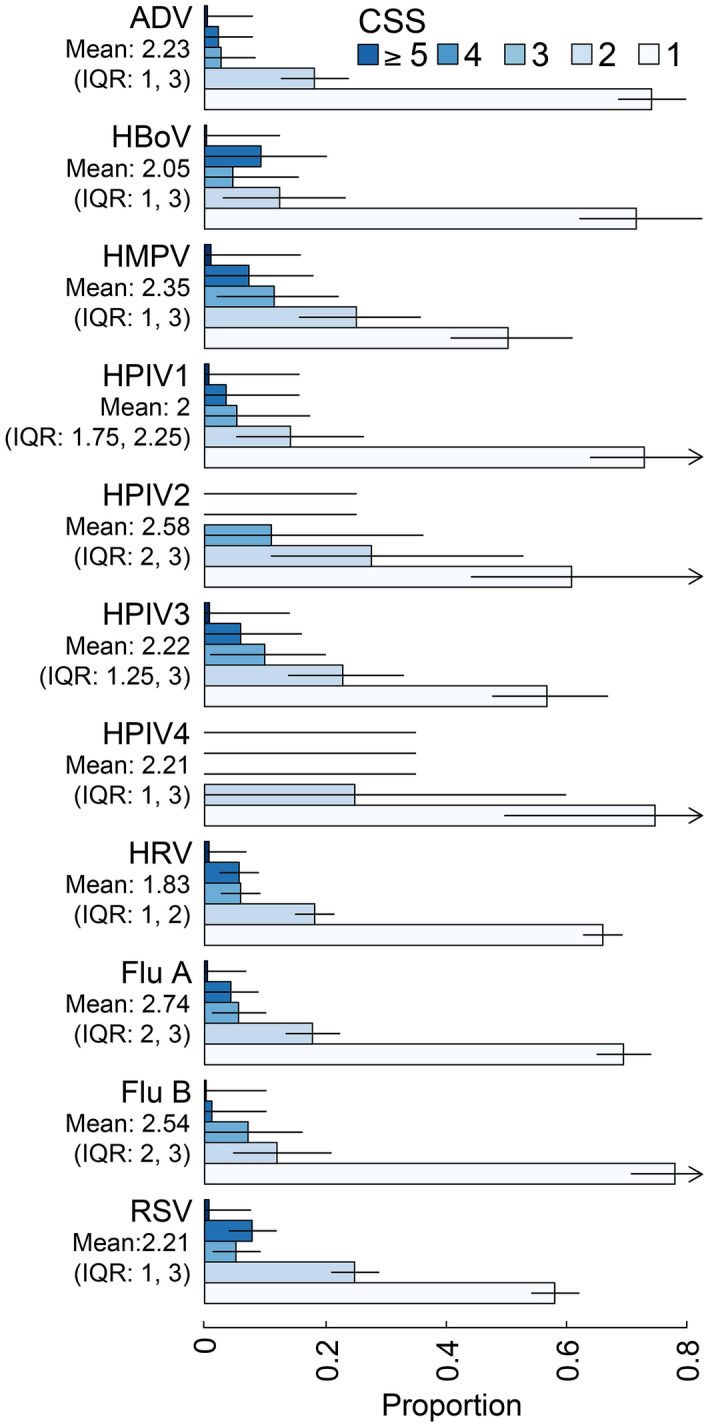
Distribution of CSS by virus. Figure shows the distributions (counts) of CSS by virus. Multinomial confidence intervals are calculated using the method of Sison and Graz^23^, as implemented in the R package MultinomialCI^24^. *ADV* Adenovirus, *HBoV* Human bocavirus, *HMPV* Human metapneumoviruses, *HPIV-1, -2, -3, -4* Human parainfluenza viruses 1–4, *HRV* Human rhinovirus, *Flu A* influenza A, *Flu B* influenza B, *RSV* Respiratory syncytial virus.

CSS ranged from a mean of 1.83 (IQR 1, 2) for HRV to 2.74 (IQR 2, 3) for Flu A (Fig. 2). Tables 3 and 4 show model estimates of the odds of being a severe case by each virus and the relative contribution of each virus to the CSS, respectively. After adjusting for various potential confounding variables, HMPV and RSV infection were 2.13 (95% CI 1.28, 3.44, $p = 0.003$) and 1.47 (95% CI 1.1, 1.95, $p = 0.009$) times more likely to be severe infections than other viruses, respectively. ADV on the other hand was associated with 55% (OR: 0.45 [95% CI 0.26, 0.73, $p = 0.003$]) lower risk of being a severe case. The other viruses were not individually associated with being a severe case. Sensitivity analyses with higher and lower cutoff for severity showed qualitatively similar trends, with ADV associated with lower odds of severity, and HMPV, HPIV3, and RSV with higher odds of severity (see Supplementary Tables 1 & 2). Flu A was negatively associated with wheezing (OR 0.68, 95% CI 0.53, 0.86, p = 0.001), while HMPV, HPIV3, and RSV were positively associated with wheezing (OR 1.85, 95% CI 1.17, 2.96, p = 0.009; 2.11, 95% CI 1.34, 3.34, p = 0.001; 1.63, 95% CI 1.31, 2.04, p < 0.001; see Supplementary Tables 3 and 4). Assessing severity across viruses without wheezing were similar to the main analyses with HMPV and RSV being associated with higher odds of being severe (OR 1.83, 95% CI 0.95, 3.27, p = 0.05; 1.82, 95% CI 1.29, 2.56, p < 0.001).

HMPV, HPIV3, and RSV were associated with higher CSS scores, with an additional 0.47 points (95% CI 0.22, 0.72, $p < 0.001$), 0.31 points (95% CI 0.06, 0.56, $p = 0.016$), and 0.3 (95% CI 0.18, 0.43, $p < 0.001$), respectively. As above, ADV was associated with 0.2 fewer points (− 0.2, [95% CI − 0.38, − 0.02, $p = 0.033$]); as was Flu B, − 0.28 (95% CI − 0.55, 0.0, $p = 0.05$). No other viruses were associated with additional or fewer points.

**Table 3:** Clinical severity by virus and number of detections.

	Total detections	Severe cases (%)	Univariate OR (95% CI, p)	Multivariate OR (95% CI, p)
ADV	219	16 (7.3%)	**0.45 (0.26, 0.73, p = 0.003)**	**0.43 (0.23, 0.76, p = 0.007)**
HBoV	64	10 (15.6%)	1.11 (0.53, 2.09, p = 0.773)	1.15 (0.54, 2.25, p = 0.697)
HMPV	95	23 (24.2%)	**1.95 (1.18, 3.1, p = 0.006)**	**2.13 (1.28, 3.44, p = 0.003)**
HPIV1	56	7 (12.5%)	0.85 (0.35, 1.76, p = 0.687)	0.74 (0.25, 1.72, p = 0.527)
HPIV2	18	2 (11.1%)	0.74 (0.12, 2.62, p = 0.693)	0.82 (0.13, 3.02, p = 0.794)
HPIV3	100	20 (20%)	1.51 (0.89, 2.44, p = 0.106)	1.51 (0.86, 2.53, p = 0.133)
HRV	819	126 (15.4%)	1.11 (0.89, 1.38, p = 0.344)	1.09 (0.85, 1.38, p = 0.487)
Flu A	390	48 (12.3%)	0.82 (0.59, 1.11, p = 0.217)	0.81 (0.57, 1.12, p = 0.211)
Flu B	83	8 (9.6%)	0.63 (0.28, 1.23, p = 0.217)	0.85 (0.37, 1.7, p = 0.662)
RSV	633	106 (16.7%)	1.25 (0.99, 1.58, p = 0.06)	**1.47 (1.1, 1.95, p = 0.009)**
PCR Neg.	1316	179 (13.6%)	Ref.	Ref.
1 detection	1711	261 (15.3%)	**1.22 (1.04, 1.42, p = 0.012)**	1.13 (0.91, 1.42, p = 0.273)
2 detections	326	38 (11.7%)	1.15 (0.88, 1.48, p = 0.301)	0.94 (0.62, 1.38, p = 0.757)
$\ge 3$ detections	42	10 (23.8%)	1.25 (0.64, 2.34, p = 0.496)	**2.65 (1.17, 5.57, p = 0.013)**

Table shows counts of hospitalizations with detections of Adenovirus (ADV), Bocavirus (HBoV), Human metapneumovirus (HMPV), H. parainfluenza 1 (HPIV1), H. parainfluenza 2 (HPIV2), H. parainfluenza 3 (HPIV3), Influenza A, Influenza B, Rhinovirus (HRV), and Respiratory syntitial virus (RSV), with the number of severe cases (defined as CSS $> 3$) for each virus. HPIV4 and H. coronavirus had no severe cases. The final two columns show the odds ratio for being a severe case (both mono- and coinfected) for the univariate model (virus only) as well as the multivariate model (adjusted for age, sex, smoking inside the child’s home, SES, calendar year, month of year, daycare attendance, and breastfeeding status). The comparator is all other cases without the focal virus. The bottom part of the table shows contributions to CSS by 1, 2, or $\ge 3$ virus detections as compared to PCR negative patients.

Estimates displayed in bold face indicate statistical significance (p<0.05).

**Table 4:** Contributions to CSS by virus and number of virus detections.

	Univariate Change in CSS (95% CI)	Multivariate Change in CSS (95% CI)
ADV	**− 0.27 (95% CI: − 0.45, − 0.1, p = 0.002)**	**− 0.2 (95% CI: − 0.38, − 0.02, p = 0.033)**
HBoV	− 0.08 (95% CI: − 0.39, 0.24, p = 0.64)	0 (95% CI: − 0.33, 0.33, p = 0.997)
HMPV	**0.44 (95% CI: 0.18, 0.7, p < 0.001)**	**0.47 (95% CI: 0.22, 0.72, p < 0.001)**
HPIV1	− 0.1 (95% CI: − 0.43, 0.24, p = 0.579)	− 0.06 (95% CI: − 0.41, 0.28, p = 0.711)
HPIV2	− 0.09 (95% CI: − 0.68, 0.51, p = 0.774)	0.01 (95% CI: − 0.59, 0.62, p = 0.966)
HPIV3	**0.28 (95% CI: 0.02, 0.53, p = 0.032)**	**0.31 (95% CI: 0.06, 0.56, p = 0.016)**
HPIV4	− 0.31 (95% CI: − 1.57, 0.95, p = 0.63)	0.14 (95% CI: − 1.25, 1.53, p = 0.842)
HRV	0.06 (95% CI: − 0.04, 0.16, p = 0.245)	0.03 (95% CI: − 0.07, 0.14, p = 0.534)
Flu A	− 0.11 (95% CI: − 0.25, 0.02, p = 0.098)	− 0.12 (95% CI: − 0.25, 0.02, p = 0.086)
Flu B	**− 0.49 (95% CI: − 0.77, − 0.21, p < 0.001)**	**− 0.28 (95% CI: − 0.55, 0, p = 0.05)**
RSV	**0.29 (95% CI: 0.18, 0.4, p < 0.001)**	**0.3 (95% CI: 0.18, 0.42, p < 0.001)**
PCR Neg.	Ref.	Ref.
1 detection	**0.13 (0.04, 0.22, p = 0.0055)**	0.11 (0.01, 0.2, p = 0.0243)
2 detections	0.04 (− 0.11, 0.2, p = 0.5759)	0.07 (− 0.09, 0.23, p = 0.3814)
$\ge$ 3 detections	0.31 (− 0.08, 0.71, p = 0.1194)	**0.51 (0.1, 0.92, p = 0.0157)**

Table shows differences in CSS by virus detected estimated by regression with CSS as the outcome with viral detections as predictors. The first column is univariate (virus only) model, the second is the multivariate model (adjusted for age, sex, smoking inside the child’s home, SES, calendar year, month of year, daycare attendance, and breastfeeding status). For example, HMPV is associated with 0.51 (95% CI 0.22, 0.80, $p<0.001$) higher CSS adjusted for the confounders. The bottom part of the table shows contributions to CSS by 1, 2, or $\ge 3$ virus detections as compared to PCR negative patients. *ADV* Adenovirus, *HBoV* Human bocavirus, *HMPV* Human metapneumoviruses, *HPIV-1, -2, -3, -4* Human parainfluenza viruses 1–4, *HRV* Human rhinovirus, *Flu A* influenza A, *Flu B* influenza B, *RSV* respiratory syncytial virus.

Estimates displayed in bold face indicate statistical significance (p<0.05).

### Severity in coinfections

In general, individuals with single and multiple viral detections had similar proportions of severe cases (0.13 [95% CI 0.1, 0.17] and 0.15 [95% CI 0.14, 0.17], two-sided proportion test: $p = 0.32$) and mean CSS (2.37 (95% CI 2.3, 2.43) and 2.31 (95% CI 2.18, 2.44), Wilcoxon signed rank: $p = 0.61$). Comparing individuals with no detections to individuals with multiple detections, we see nearly identical proportions of severe cases, with 0.13 [95% CI 0.098, 0.169] and 0.136 [95% CI 0.118, 0.156] of PCR negatives and multiple PCR positives severe, respectively; and no difference in mean CSS score (2.23 [95% CI 2.17, 2.3] and 2.31 [95% CI 2.18, 2.44],Wilcoxon signed rank: $p = 0.14$)). PCR negative individuals did not have a smaller proportion of severe cases than individuals with one PCR detection (0.14 [95% CI 0.12, 0.16] vs. 0.15 [95% CI 0.14, 0.17], two-sided proportion test: $p = 0.22$), and one PCR detection had a higher CSS (2.37 [95% CI 2.3, 2.43] vs. 2.23 [95% CI 2.17, 2.3]; Wilcoxon signed rank: $p = 0.001$), which may or may not have clinical significance. We do see higher odds of severity and CSS score in individuals with at least 3 detections with nearly 3-fold higher adjusted OR of being severe (OR 2.65 [95% CI 1.17, 5.57, p = 0.013]) and 0.51 (95% CI 0.1, 0.92, p = 0.016) higher CSS scores. However, due to the small numbers of patients with $\ge 3$ concurrent detections, we test for sparse data bias using Bayesian logistic regressions with informative priors. We find a diminishing of both the effect size and statistical significance (OR 1.63; 95% credible interval: 0.85, 2.97, p = 0.05), though the effect is still suggestive of these infections being more severe (see Supplementary Table 5 and Figures S1 and S2). Findings in children with no underlying conditions are qualitatively similar to those presented here (Supplementary Table 6).

**Figure 3:**
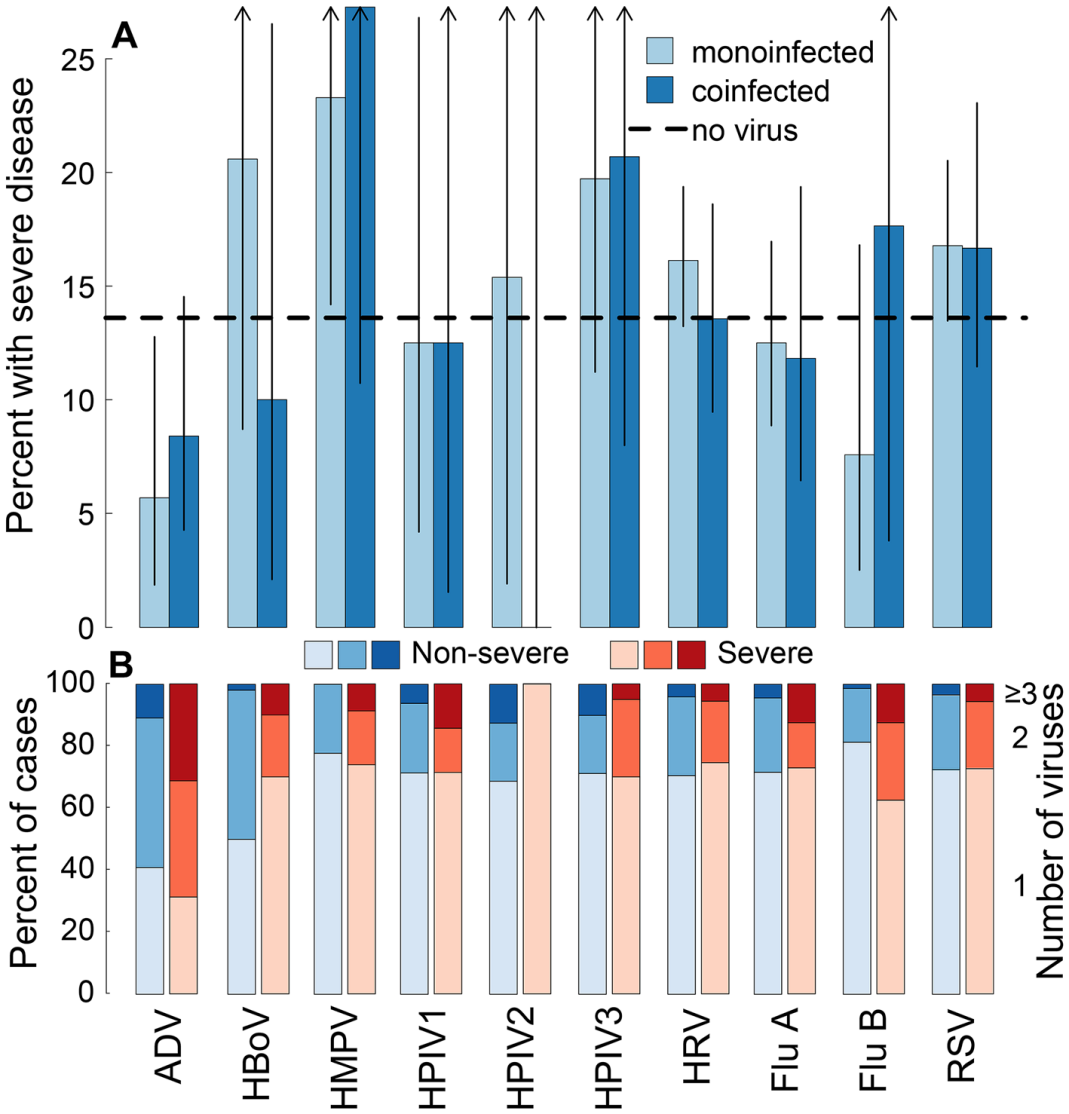
Percent of patients with severe disease by virus for no-, mono-, and multiple-infections and percent of severe and non-severe cases by virus and number of viruses detected in the NP sample. Panel (**A**) shows the percent of patients suffering severe disease (CSS $>3$) by virus and whether the patient was PCR negative (dashed black line), PCR positive for 1 virus (light blue), or PCR positive for more than 1 virus (dark blue). Line segments indicate 95% binomial confidence intervals. Panel (**B**) show the percent of severe and non-severe patients by virus and PCR positive for 1, 2, or $\ge 3$ viruses, respectively. *ADV* Adenovirus, *HBoV* Human bocavirus, *HMPV*Human metapneumoviruses, *HPIV-1, -2, -3, -4* Human parainfluenza viruses 1–4, *HRV* Human rhinovirus, *Flu A* influenza A, *Flu B* influenza B, *RSV* respiratory syncytial virus.

**Figure 4:**
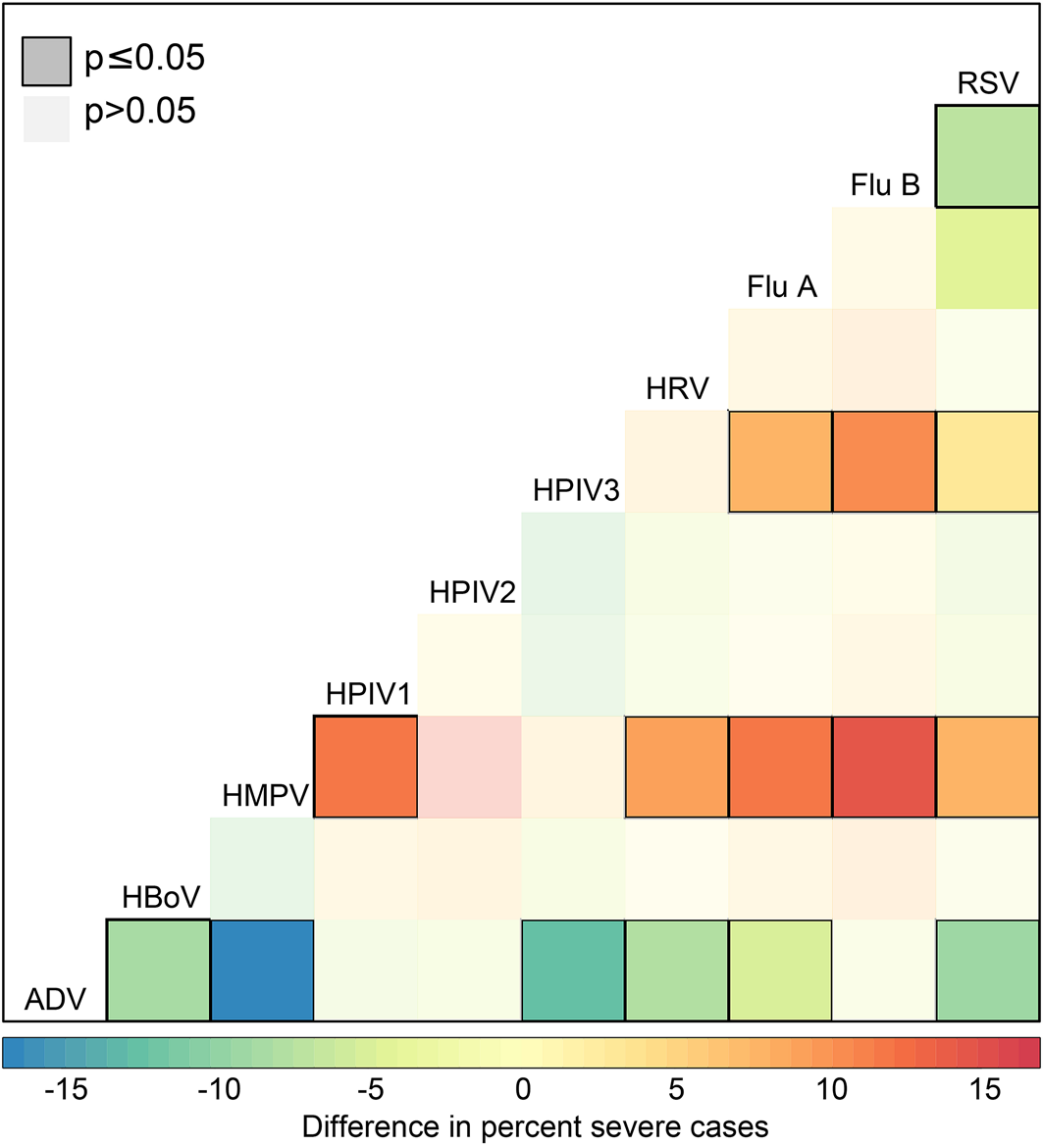
Difference in proportion severe cases between all viruses. Figure shows the difference in percent of patients suffering severe disease (CSS $>3$) by virus with each other virus. Dark squares outlined in black indicate significant differences between percents as tested by a one-sided proportion test. For example, the bottom row indicates the proportion severe ADV cases is significantly lower than proportions of severe cases from HBoV, HMPV, HPIV3, HRV, Flu A, and RSV. This is comparing mono- and multiply-infected individuals. *ADV*Adenovirus, *HBoV* Human bocavirus, *HMPV*Human metapneumoviruses, *HPIV-1, -2, -3, -4* Human parainfluenza viruses 1–4, *HRV* Human rhinovirus, *Flu A* influenza A, *Flu B* influenza B, *RSV* respiratory syncytial virus.

Examination of proportions of severity by number of PCR detections by individual viruses shows similar patterns across viruses (Fig. 3). There are no statistically significant differences in the proportion of severe individuals in mono- or multiply-infected individuals by virus or in PCR negative individuals.

Though not statistically significant, individuals with Flu B coinfections were more severe than mono-infected Flu B individuals (0.18 [95% CI 0.04, 0.43] vs. 0.08 [95% CI 0.03, 0.17]; one-sided proportion test: $p = 0.07$) and PCR negative individuals (0.29 [0.08, 0.58] and 0.13 [0.11, 0.15]; one-sided proportion test: $p = 0.09$). Mean CSS scores were similar between Flu B coinfections, monoinfections, and PCR negative individuals (2.29 [95% CI 1.34, 3.23], 1.8 [95% CI 1.5, 2.09]], and 2.2 [95% CI 2.12, 2.28], respectively). While the proportion of severe cases is similar for single and coinfections with ADV (0.07 [95% CI 0.02, 0.17] and 0.05 [95% CI 0.02, 0.12]; two-sided proportion test: $p = 0.98$), the proportion of severe cases in mono- or coinfected ADV is lower than PCR negative individuals (0.05 [95% CI 0.02, 0.12] and 0.13 [95% CI 0.11, 0.15]; one-sided proportion test: $p = 0.026$).

Finally, comparing the proportion of severe cases virus to virus, we see ADV as consistently having lower proportions of severe cases as compared to the other viruses, and HMPV and RSV as consistently having higher proportions of severe cases (Fig. 4).

## Discussion

Here we investigate the range of clinical severity for patients singly, multiply and not infected with a group of 13 commonly circulating viruses in Nha Trang, Vietnam. Largely we find no difference in severity by multiplicity of viral infections and little differences in clinical severity between patients carrying different viruses. Of note, there is suggestion that infection with more than 2 viruses is more severe than infection with 0, 1, or 2 viruses. Also, detection of HMPV and RSV—either as as monoinfection or as part of a coinfection—was associated with increased risk of being severe, this association being robust to inclusion of wheezing. Finally, we find infection with ADV to be consistently associated with lower risk of severity. While clinically it is difficult or impossible to distinguish between the various infecting pathogen, if RSV or HMPV virus is suspected, PCR testing for confirmatory diagnosis and for detection of multiple coinfecting viruses would be fruitful to assess the probability of whether the patient’s disease course is going to be severe. If PCR testing reveals the presence of the other viruses, however, the clinician may expect a less severe clinical course.

Previous work has found increased severity with RSV and HMPV coinfection. Semple et al.^6^ found dual infection with RSV and HMPV (as detected using NP aspirate) to be strongly associated with 11-fold higher risk of admission to the pediatric intensive care unit as compared to RSV monoinfection, but a non-significant increase in risk of severe disease (defined as admission to the hospital and use of supplemental oxygen). Of our 3403 enrolled patients only 2 had RSV/HMPV coinfection, 1 of which had a CSS of 5. Using data from the same study site but limited to April 2007 through to March 2010, Yoshida et al.^5^ found RSV to be the leading pathogen associated with ARI hospitalization and RSV and HMPV to be associated with LRTI. Harada et al.^27^ found 2.95- and 2.4-fold increases in odds of nonpneumonic ARI and RCP as compared to mild disease, respectively, in individuals positive for RSV either singly or as part of a coinfection, and a nearly 5-fold increase in odds of nonpneumonic ARI vs. mild disease in coinfected RSV individuals. We consistently found RSV to be associated with higher odds of severity and CSS.

Several studies have indicated that the clinical symptoms and severity of HMPV infections are similar to that of RSV^8,9,28,29^. Indeed, both viruses are of the family *Paramyxoviridae* and recent work has identified cross-reactive neutralizing antibodies to the fusion proteins of both HMPV and RSV^30–32^. While we find a higher proportion of severe cases in HMPV vs. RSV infected individuals (0.24 vs. 0.17, $p = 0.04$), we find no statistical difference between the odds of severe disease or raw CSS, though the point estimates suggest HMPV may be more severe. Future work could further examine differences in HMPV and RSV cases.

Surprisingly, we find a reduced severity with ADV infection as compared to the other viruses in our study. ADV infection severity is under-explored and open questions remain about the role of ADV coinfection and clinical severity^12^. Martin et al.^33^ found over 50% of 103 identified coinfected samples tested positive for ADV and that ADV coinfections had a significantly lower viral load than ADV monoinfections. ADV is often carried asymptomatically and may have persistent viral shedding^34,35^, perhaps individuals admitted for ARI had some other etiology than the present ADV infection. It is known that different ADV types are associated with different clinical severity^36,37^. For example, the 9 identified ADV types in a cohort of Korean children varied in clinical severity from 0 severe cases for ADV-4 and -6 to 23 (25%) for ADV-7^36^. As we do not have information on ADV type in our hospitalized patients, it may be that dominant type in Southern Vietnam is less severe. These serotype differences may also explain the results of Mazur et al.^38^ which found 3.4-fold increase in the odds of severe disease for ADV coinfections. Identification of the circulating ADV types in Vietnam would further elucidate our observed patterns.

Recent meta-analyses have found no increased severity in ARI for coinfected individuals^2–4^. We find a suggestion of increased severity only in individuals with 3 or more detections. While this outcome was rare (42 individuals, 1.2%), it may be that the presence of 3 or more viruses puts added pressure on the immune system resulting in a more intense response, or perhaps those children had underlying conditions to predispose them to multiple coinfections. We acknowledge that this finding cannot be ruled out that this result is a statistical artifact due to small data bias, though the point estimate is for it being more severe. Future work could more fully evaluate these multiply-infected individuals for salient features of severity, and evaluation of individuals in other settings would help explore this finding more.

Due to the nature of the study we are not able to determine the order of infection for coinfected individuals. Longitudinal study of a cohort of individuals with multiple NP swabs taken over time would allow understanding of the temporal interaction of these respiratory viruses. It would be interesting to see if infection with RSV or HMPV modifies future infection risk from other viruses. Further limitations of the present study include the fact that this study used hospital-based surveillance, necessarily presenting the most ill children which may have differing underlying risk for having severe disease. In addition, this study does not allow comparison with outpatient severity. However our focus was on elucidating the potential severity of children hospitalized for ARI. And, by the time the individual is hospitalized, a pathogen may have been cleared, and not detected in the NP swab. It may be that infection with one pathogen enhanced the severity of a subsequent infection leading to hospitalization with the first infection cleared. We are unable to detect enhancing infections of this type, and hence may under-estimate the clinical role of viral co-infection. We do not have information on subsequent admission to the pediatric intensive care unit, nor use of high flow nasal cannula or continuous positive airway pressure support in the pediatric intensive care unit. Subsequent studies should include these indicators as early prediction of use of these medically serious and expensive outcomes would be beneficial to clinical care. We acknowledge that in clinical practice it is not uncommon to identify infants with bronchiolitis and normal chest X rays. It is why we have other clinical parameters in the scoring system and our main outcome was being a severe case not a positive chest X ray. Although we have not adjusted for multiple testing here, ours is an exploratory study to identify risk factors for severity which will require future work to elucidate. In addition our main study conclusions are robust to multiple cutoffs defining our main endpoint as well as in a Bayesian regression setting. Finally, we are not considering bacterial agents as the potential etiology of hospital admissions. It may be that observed similarities in severity across viruses is an indication that viral detection has little to do with the disease episode and that other etiologies are at play. Future work should explore the role of bacteria in disease etiologies in this setting.

Our work identifies risk of severe outcomes by specific viruses in hospitalized individuals and lends to the suggestion of PCR screening individuals as they enter the hospital. Future work could build on these observed patterns and test specific mechanisms for differences in disease severity. It could also examine risk of coinfection from contact clustering^39,40^, or the impact of environmental factors on disease risk. Our study provides evidence that while in some cases multiple viral infection may provide a perfect storm causing serious disease, viral coinfection as a cause of mild or severe ARI is generally uncommon both in its absolute frequency and in its risk enhancement for severe disease compared to a single virus infection.

## Supplementary Information

Supplementary Information
