# Supplementary material for: Differences in clinical severity of respiratory viral infections in hospitalized children
Source: Sci Rep. 2021 Mar 4;11:5163. doi: 10.1038/s41598-021-84423-2 (PMC7933285; doi:10.1038/s41598-021-84423-2)
Supplement: Supplementary file 1 — Supplementary Information [file 41598_2021_84423_MOESM1_ESM.pdf]

Supporting Information for Differences in clinical severity of respiratory viral infections in hospitalized children

Benjamin M Althouse, Stefan Flasche, Michiko Toizumi, Hien-Anh Thi Nguyen, Hien Minh Vo, Le Nhat Minh, Masahiro Hashizume, Koya Ariyoshi, Dang Duc Anh, Gail L. Rodgers, Keith P. Klugman, Hao Hu, Lay-Myint Yoshida

Different cutoff for severity

Supplementary Tables 1 & 2 show the results of the logistic regression with a lower and higher severity score cutoff.

	Total detections	Severe cases (%)	Univariate OR (95% CI, p)	Multivariate OR (95% CI, p)
ADV	219	56 (25.6%)	**0.67 (0.49, 0.91, p = 0.012)**	0.77 (0.54, 1.09, p = 0.148)
HBoV	64	18 (28.1%)	0.78 (0.44, 1.33, p = 0.375)	0.88 (0.48, 1.56, p = 0.668)
HMPV	95	47 (49.5%)	**2 (1.33, 3.02, p < 0.001)**	**2.08 (1.35, 3.19, p < 0.001)**
HPIV1	56	15 (26.8%)	0.73 (0.39, 1.29, p = 0.298)	0.82 (0.41, 1.53, p = 0.548)
HPIV2	18	7 (38.9%)	1.28 (0.47, 3.25, p = 0.616)	1.64 (0.57, 4.52, p = 0.342)
HPIV3	100	43 (43%)	**1.53 (1.02, 2.28, p = 0.038)**	**1.74 (1.12, 2.67, p = 0.012)**
HPIV4	4	1 (25%)	0.67 (0.03, 5.22, p = 0.726)	1.27 (0.06, 13.6, p = 0.846)
HRV	819	276 (33.7%)	1.02 (0.87, 1.21, p = 0.788)	0.96 (0.8, 1.15, p = 0.632)
Flu A	390	118 (30.3%)	0.85 (0.68, 1.07, p = 0.174)	0.83 (0.65, 1.06, p = 0.146)
Flu B	83	18 (21.7%)	**0.55 (0.31, 0.91, p = 0.025)**	0.82 (0.46, 1.39, p = 0.481)
RSV	633	264 (41.7%)	**1.56 (1.31, 1.87, p < 0.001)**	**1.68 (1.36, 2.09, p < 0.001)**
PCR Neg.	1316	405 (30.8%)	Ref.	Ref.
1 detection	1711	601 (35.1%)	**1.22 (1.04, 1.42, p = 0.012)**	**1.21 (1.02, 1.43, p = 0.026)**
2 detections	326	110 (33.7%)	1.15 (0.88, 1.48, p = 0.301)	1.21 (0.91, 1.6, p = 0.178)
≥ 3 3 detections	42	15 (35.7%)	1.25 (0.64, 2.34, p = 0.496)	1.68 (0.82, 3.38, p = 0.148)

Supplementary Table 1: **Clinical severity by virus – cutoff 2.** Table is identical to Table 3 in the main text, with a cutoff for being a severe case of 2, i.e., a severe case is a patient with CSS > 2.

	Total detections	Severe cases (%)	Univariate OR (95% CI, p)	Multivariate OR (95% CI, p)
ADV	219	10 (4.6%)	**0.51 (0.25, 0.92, p = 0.039)**	0.64 (0.3, 1.22, p = 0.212)
HBoV	64	7 (10.9%)	1.35 (0.56, 2.8, p = 0.455)	1.48 (0.59, 3.18, p = 0.352)
HMPV	95	12 (12.6%)	1.61 (0.83, 2.87, p = 0.131)	1.82 (0.92, 3.31, p = 0.064)
HPIV1	56	4 (7.1%)	0.84 (0.25, 2.07, p = 0.739)	0.86 (0.21, 2.43, p = 0.808)
HPIV3	100	10 (10%)	1.23 (0.59, 2.27, p = 0.55)	1.26 (0.57, 2.45, p = 0.532)
HRV	819	77 (9.4%)	1.19 (0.9, 1.56, p = 0.219)	1.19 (0.88, 1.6, p = 0.252)
Flu A	390	26 (6.7%)	0.76 (0.49, 1.13, p = 0.199)	0.75 (0.47, 1.14, p = 0.199)
Flu B	83	2 (2.4%)	0.27 (0.04, 0.85, p = 0.065)	0.41 (0.07, 1.36, p = 0.226)
RSV	633	73 (11.5%)	**1.58 (1.18, 2.08, p = 0.002)**	**1.84 (1.29, 2.62, p < 0.001)**
PCR Neg.	1316	96 (13.6%)	Ref.	Ref.
1 detection	1711	161 (15.3%)	**1.32 (1.02, 1.72, p = 0.039)**	**1.36 (1.02, 1.84, p = 0.04)**
2 detections	326	21 (11.7%)	0.88 (0.52, 1.4, p = 0.592)	1.07 (0.62, 1.77, p = 0.798)
≥ 3 3 detections	42	6 (23.8%)	2.12 (0.79, 4.8, p = 0.098)	**2.79 (1, 6.71, p = 0.031)**

Supplementary Table 2: **Clinical severity by virus – cutoff 4.** Table is identical to Table 3 in the main text, with a cutoff for being a severe case of 4, i.e., a severe case is a patient with CSS > 4.

Wheezing results

Supplementary Table 3 shows the associations between virus isolations and wheezing, and Supplementary Table 4 the results of the logistic regression with severity scores calculated without wheezing.

	Univariate OR (95% CI, p)	Multivariate OR (95% CI, p)
ADV	**0.74 (0.56, 0.97, p = 0.032)**	1.04 (0.75, 1.43, p = 0.816)
HBoV	0.71 (0.42, 1.17, p = 0.189)	0.87 (0.48, 1.55, p = 0.631)
CoV	0.8 (0.24, 2.5, p = 0.7)	1.14 (0.29, 3.91, p = 0.841)
HMPV	**1.7 (1.13, 2.6, p = 0.012)**	**1.85 (1.17, 2.96, p = 0.009)**
HPIV1	0.62 (0.35, 1.06, p = 0.085)	0.8 (0.43, 1.44, p = 0.462)
HPIV2	0.56 (0.19, 1.44, p = 0.243)	0.7 (0.23, 2.02, p = 0.522)
HPIV3	**1.5 (1.01, 2.25, p = 0.048)**	**2.11 (1.34, 3.34, p = 0.001)**
HPIV4	1.12 (0.13, 9.33, p = 0.911)	3.23 (0.29, 72.12, p = 0.351)
HRV	**1.19 (1.02, 1.4, p = 0.028)**	1.17 (0.98, 1.4, p = 0.089)
Flu A	**0.8 (0.64, 0.99, p = 0.039)**	**0.68 (0.53, 0.86, p = 0.001)**
Flu B	**0.35 (0.2, 0.57, p < 0.001)**	0.61 (0.35, 1.03, p = 0.075)
RSV	**1.72 (1.44, 2.05, p < 0.001)**	**1.63 (1.31, 2.04, p < 0.001)**

Supplementary Table 3: **Associations of virus detections with wheezing.** Table The final two columns show the odds ratio for being a severe case (both mono- and coinfected) for the univariate model (virus only) as well as the multivariate model (adjusted for age, sex, smoking inside the child's home, SES, calendar year, month of year, daycare attendance, and breastfeeding status).

	Total detections	Severe cases (%)	Univariate OR (95% CI, p)	Multivariate OR (95% CI, p)
ADV	219	12 (5.5%)	0.57 (0.3, 0.99, p = 0.064)	0.59 (0.28, 1.12, p = 0.14)
HBoV	64	7 (10.9%)	1.25 (0.52, 2.58, p = 0.582)	1.37 (0.55, 2.93, p = 0.456)
HMPV	95	13 (13.7%)	1.63 (0.86, 2.87, p = 0.108)	**1.83 (0.95, 3.27, p = 0.05)**
HPIV1	56	4 (7.1%)	0.78 (0.23, 1.91, p = 0.628)	0.79 (0.19, 2.2, p = 0.691)
HPIV2	18	0 (0%)	0 (NA, 1888.6, p = 0.969)	0 (NA, 13026.34, p = 0.97)
HPIV3	100	11 (11%)	1.26 (0.63, 2.29, p = 0.475)	1.3 (0.62, 2.48, p = 0.447)
HRV	819	81 (9.9%)	1.15 (0.88, 1.5, p = 0.298)	1.15 (0.85, 1.53, p = 0.362)
Flu A	390	26 (6.7%)	0.7 (0.45, 1.04, p = 0.091)	0.68 (0.43, 1.04, p = 0.086)
Flu B	83	2 (2.4%)	**0.25 (0.04, 0.78, p = 0.05)**	0.36 (0.06, 1.19, p = 0.164)
RSV	633	77 (12.2%)	**1.54 (1.16, 2.02, p = 0.002)**	**1.82 (1.29, 2.56, p < 0.001)**
PCR Neg.	1316	42 (8.1%)	Ref.	Ref.
1 detection	1711	56 (10%)	1.12 (0.92, 1.36, p = 0.272)	1.03 (0.64, 1.65, p = 0.909)
2 detections	326	11 (6.7%)	0.77 (0.53, 1.1, p = 0.161)	1.3 (0.6, 2.62, p = 0.477)
>2 detections	42	2 (14.3%)	1.72 (0.79, 3.44, p = 0.142)	1.97 (0.31, 7.06, p = 0.373)

Supplementary Table 4: **Clinical severity by virus, excluding wheezing.** Table is identical to Table 3 in the main text, with the severity score not including wheezing. HPIV2 & 4 had 0 severe cases.

Bayesian logistic regression

Supplementary Table 5 and Figures S1 & S2 present the results of Bayesian logistic regressions to account for potential sparse data bias [1]. Prior means for the β coefficients were assumed to be multivariate-normal distributed with mean 0 and prior precision for β of 5 under a multivariate normal prior. Markov Chains were run for a 50,000 burn-in period and 100,000 samples with thinning every 10 samples were used for inference. Analyses were conducted using the `MCMClogit` function in the R package `MCMCpack` [2].

	Total detections	Severe cases (%)	Univariate OR (95% CI, p)	Multivariate OR (95% CI, p)
ADV	219	16 (7.3%)	0.54 (0.35, 0.81, p = 0.999)	0.53 (0.33, 0.81, p = 0.998)
HBoV	64	10 (15.6%)	1.04 (0.6, 1.75, p = 0.442)	1.09 (0.61, 1.89, p = 0.4)
HMPV	95	23 (24.2%)	**1.63 (1.05, 2.48, p = 0.014)**	**1.73 (1.09, 2.69, p = 0.01)**
HPIV1	56	7 (12.5%)	0.89 (0.49, 1.57, p = 0.645)	0.79 (0.45, 1.44, p = 0.761)
HPIV2	18	2 (11.1%)	0.91 (0.44, 1.9, p = 0.599)	0.94 (0.42, 2.02, p = 0.568)
HPIV3	100	20 (20%)	1.33 (0.84, 2.05, p = 0.11)	1.23 (0.81, 1.92, p = 0.172)
HRV	819	126 (15.4%)	1.08 (0.87, 1.33, p = 0.245)	1.11 (0.88, 1.41, p = 0.182)
Flu A	390	48 (12.3%)	0.82 (0.6, 1.09, p = 0.911)	0.87 (0.65, 1.21, p = 0.781)
Flu B	83	8 (9.6%)	0.74 (0.43, 1.24, p = 0.87)	0.8 (0.45, 1.28, p = 0.807)
RSV	633	106 (16.7%)	1.21 (0.95, 1.5, p = 0.057)	**1.43 (1.09, 1.88, p = 0.004)**
PCR Neg.	1316	179 (13.6%)	Ref.	Ref.
1 detection	1711	261 (15.3%)	1.07 (0.88, 1.31, p = 0.239)	1.14 (0.93, 1.42, p = 0.097)
2 detections	326	38 (11.7%)	0.81 (0.57, 1.13, p = 0.893)	0.94 (0.64, 1.36, p = 0.635)
$\geq$ 3 detections	42	10 (23.8%)	1.44 (0.78, 2.53, p = 0.114)	**1.63 (0.85, 2.97, p = 0.05)**

Supplementary Table 5: **Bayesian logistic regression.** Table is identical to Table 3 in the main text but under a Bayesian framework. Estimates are the medians with 95% credible intervals (CI).

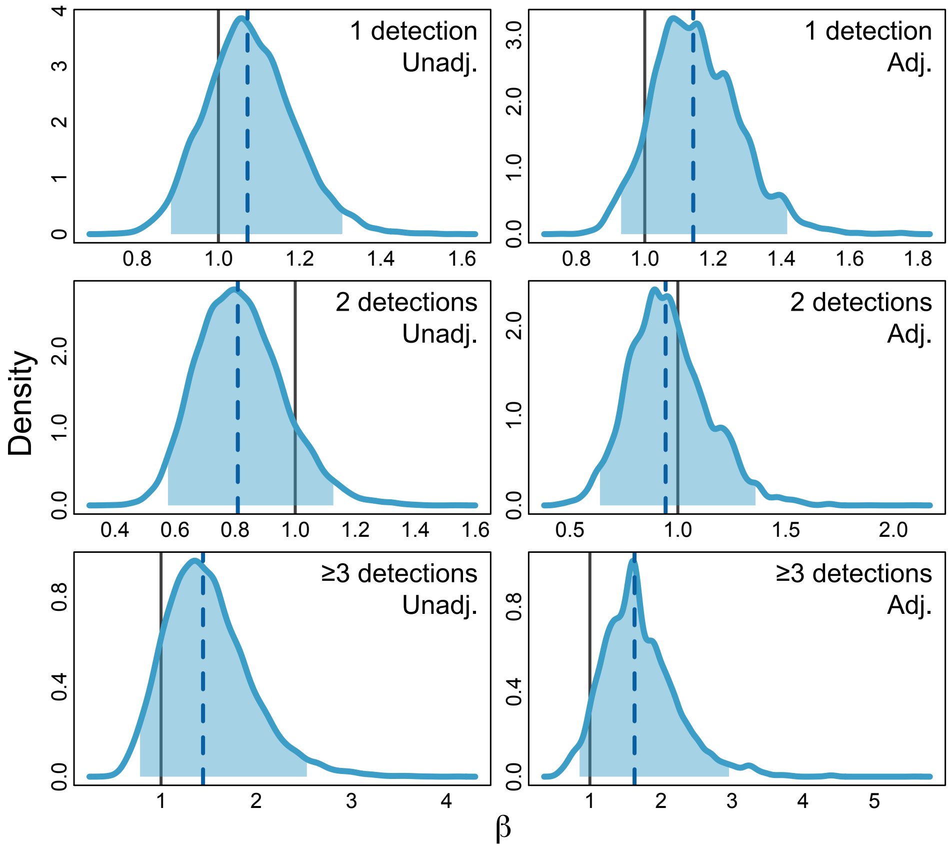

Figure S1: **Posterior density plots of coefficients from Bayesian regressions.** Figure shows the posterior densities from the Bayesian logistic regression for numbers of concurrent virus isolations. Dashed vertical lines indicate median values of β, and solid vertical lines indicate no effect ($\beta = 1$).

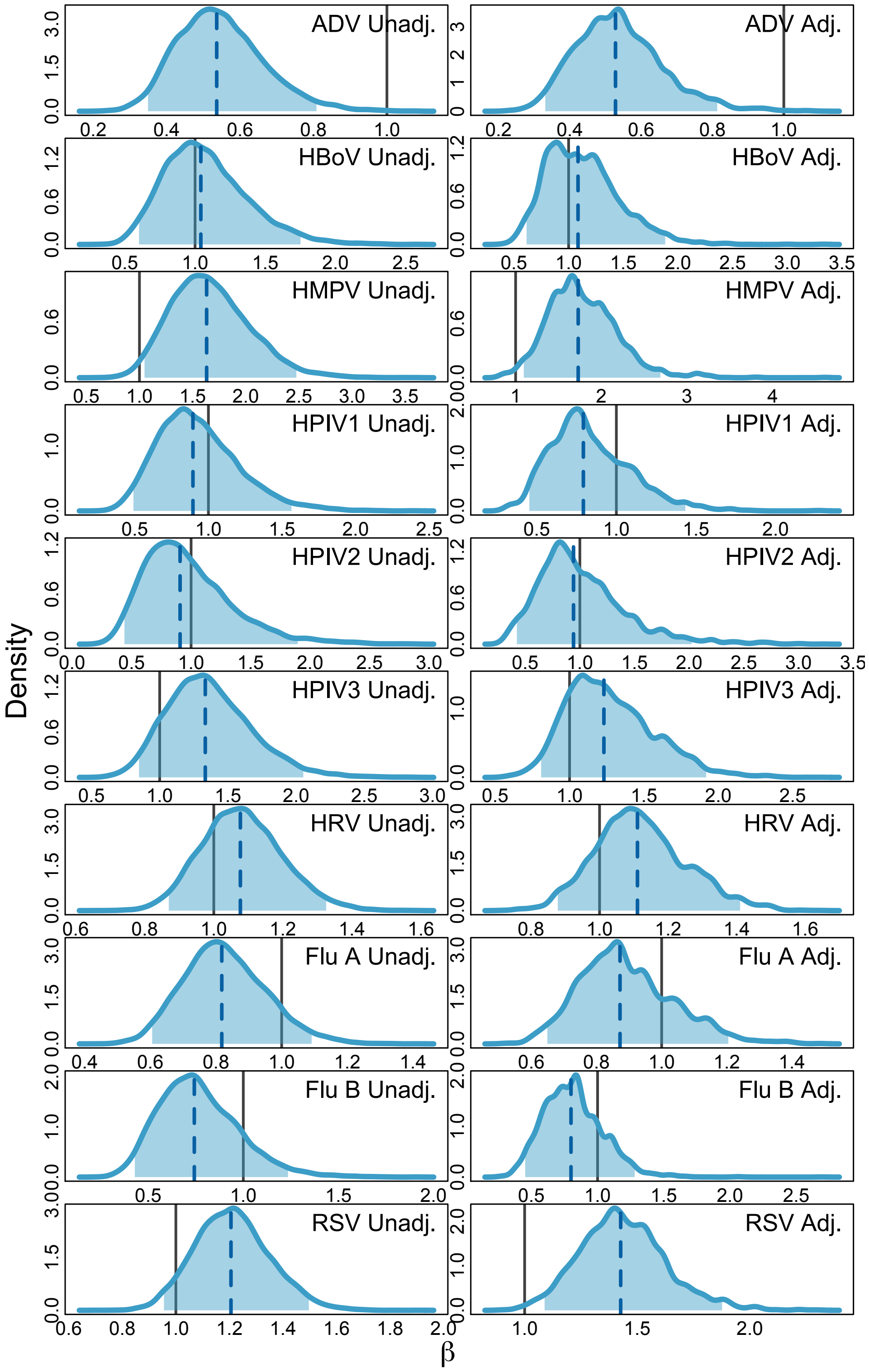

Figure S2: **Posterior density plots of coefficients from Bayesian regressions.** Figure shows the posterior densities from the Bayesian logistic regression for the virus isolations. Dashed vertical lines indicate median values of β, and solid vertical lines indicate no effect ($\beta = 1$).

No underlying conditions

	Total detections	Severe cases (%)	Univariate OR (95% CI, p)	Multivariate OR (95% CI, p)
	Total detections	Severe cases (%)	Univariate OR (95% CI, p)	Multivariate OR (95% CI, p)
ADV	128	7 (5.5%)	0.56 (0.24, 1.14, p = 0.15)	0.64 (0.22, 1.5, p = 0.356)
HBoV	32	3 (9.4%)	1.04 (0.25, 2.98, p = 0.945)	1.37 (0.31, 4.24, p = 0.63)
HMPV	32	6 (18.8%)	2.38 (0.87, 5.5, p = 0.06)	2.54 (0.9, 6.21, p = 0.054)
HPIV1	36	3 (8.3%)	0.91 (0.22, 2.59, p = 0.883)	0.37 (0.02, 1.8, p = 0.333)
HPIV2	10	1 (10%)	1.12 (0.06, 6.02, p = 0.915)	1.65 (0.09, 9.92, p = 0.646)
HPIV3	49	7 (14.3%)	1.71 (0.69, 3.64, p = 0.198)	1.16 (0.38, 2.87, p = 0.766)
HRV	392	41 (10.5%)	1.24 (0.84, 1.78, p = 0.264)	1.44 (0.92, 2.2, p = 0.098)
Flu A	198	13 (6.6%)	0.68 (0.36, 1.18, p = 0.202)	0.83 (0.42, 1.51, p = 0.568)
Flu B	58	4 (6.9%)	0.74 (0.22, 1.83, p = 0.566)	0.86 (0.25, 2.22, p = 0.779)
RSV	314	34 (10.8%)	1.28 (0.85, 1.89, p = 0.221)	**1.74 (1.01, 2.96, p = 0.042)**
PCR Neg.	727	179 (13.6%)	Ref.	Ref.
1 detection	856	261 (15.3%)	1.06 (0.84, 1.34, p = 0.625)	1.11 (0.73, 1.68, p = 0.631)
2 detections	166	38 (11.7%)	0.98 (0.65, 1.46, p = 0.94)	1.38 (0.67, 2.68, p = 0.358)
$\geq$ 3 detections	23	10 (23.8%)	0.95 (0.31, 2.43, p = 0.926)	**5.01 (1.26, 16.96, p = 0.013)**

Supplementary Table 6: **Clinical severity by virus in children with no known underlying conditions.** Table is identical to Table 3 in the main text in 1772 children with no known underlying conditions.

References

[1] S. Greenland, M. A. Mansournia, and D. G. Altman, "Sparse data bias: a problem hiding in plain sight," *bmj*, vol. 352, p. i1981, 2016.

[2] A. D. Martin, K. M. Quinn, and J. H. Park, "MCMCpack: Markov chain monte carlo in R," *Journal of Statistical Software*, vol. 42, no. 9, p. 22, 2011.
